# Strong Fe^3+^-O(H)-Pt Interfacial Interaction Induced Excellent Stability of Pt/NiFe-LDH/rGO Electrocatalysts

**DOI:** 10.1038/s41598-018-19876-z

**Published:** 2018-01-22

**Authors:** Yechuang Han, Pengfei Li, Jun Liu, Shouliang Wu, Yixing Ye, Zhenfei Tian, Changhao Liang

**Affiliations:** 1 Key Laboratory of Materials Physics and Anhui Key Laboratory of Nanomaterials and Nanotechnology, Institute of Solid State Physics, Hefei Institutes of Physical Science, Chinese Academy of Sciences, Hefei, 230031 China; 2 Department of Materials Science and Engineering, University of Science and Technology of China, Hefei, 230026 China

## Abstract

Agglomeration-triggered deactivation of supported platinum electrocatalysts markedly hinders their application in methanol oxidation reaction (MOR). In this study, graphene-supported nickel–iron layered double hydroxide (NiFe-LDH/rGO), in which Fe^3+^ was introduced to replace Ni^2+^ partially in the Ni(OH)_2_ lattice to provide stronger metal–support bonding sites, was utilized to immobilize Pt nanoparticles (NPs). Given the optimized metal–support interfacial contact (Fe^3+^-O(H)-Pt) between Pt NPs and NiFe-LDH/rGO nanosheets for Pt/NiFe-LDH/rGO electrocatalysts, the Pt/NiFe-LDH/rGO electrocatalysts displayed dramatically enhanced durability than that of Pt/Ni(OH)_2_/rGO counterpart as well as commercial Pt/C, and 86.5% of its initial catalytic activity can be maintained even after 1200 cycles of cyclic voltammetry (CV) tests during MOR. First-principle calculations toward the resultant M-O(H)-Pt (M = Fe^3+^, Ni^2+^) interfacial structure further corroborates that the NiFe-LDH nanosheets can provide stronger bonding sites (via the Fe^3+^-O(H)-Pt bonds) to immobilize Pt NPs than those of Ni(OH)_2_ nanosheets (via the Ni^2+^-O(H)-Pt bonds).

## Introduction

Graphene-supported Pt nanoparticles (NPs) have attracted sustained attention due to their distinct electrocatalytic performance in methanol oxidation reaction^1^. However, given the high surface energy, Pt NPs easily agglomerate and grow during catalytic reactions, especially for Pt NPs with a few nanometers in size^2,3^. Moreover, Pt-based catalysts are readily poisoned with chemisorbed intermediates generated during operations^4,5^. To overcome these two severe drawbacks, long-lived and anti-poisoned Pt electrocatalysts should be developed. The selection of proper supported materials and loading method are key points in constructing ideal Pt electrocatalysts^6–8^. Graphene modified with metal hydroxides or oxides is commonly selected as support to immobilize Pt NPs because graphene can transport electrons efficiently; in addition, metal hydroxides or oxides can facilitate water dissociation to generate oxygen-contained species, thereby accelerating the removal of poisoning carbonaceous species on adjacent Pt sites^9–11^.

Among a variety of metal hydroxides and oxides, layered double hydroxides (LDHs) present more advantages because of their distinct structures and highly synergetic with Pt NPs^12–15^. As a class of 2D anionic clays, LDHs possess positively charged brucite-like layers and intercalated anions^12^. Normally, the M^2+^ cations in the host structure of hydroxides are partially replaced with M^3+^ cations, and the excessive cationic charge generated by M^3+^ cations is balanced with anionic intercalation between different hydroxide layers^13^. Benefiting from the atomic-scale uniform distribution of site-specific and edge-sharing MO_6_ octahedra in the host structure, LDHs exhibit considerable potential as supports to immobilize Pt NPs due to the strong contact between metal and site-specific supports^14^. Recently, Chen and co-workers^15^ demonstrated that Pt NPs, covered with an atomic layer thickness of NiFe–LDH, are highly durable and efficient for the catalytic oxidation of carbon monoxide by taking advantage of the strong interfacial effect. Additionally, the combination of iron and nickel can significantly increase the water splitting ability by taking advantage of synergistic metal–metal interactions^16–20^. Furthermore, LDHs are more favorable toward water dissociation than that of metal oxides in alkaline electrolytes^16^. For example, NiFe–LDH nanosheets present comparable water splitting activity to that of scarce and expensive materials, such as IrO_2_ and RuO_2_^19^. Despite some achievements, two fundamental limitations which have plagued the full use of LDH supported Pt nanocatalysts still remain: (i) in the contact interfaces between Pt NPs and LDH support, which part (M^2+^O_6_ or M^3+^O_6_ octahedra) is decisive in immobilizing Pt NPs is still not clear; (ii) traditional solution reduction method, reduce Pt precursors with reductant in liquids, cannot ensure that all Pt nanosrystals directly grown on the LDH supports.

In the present work, we selected the graphene-supported NiFe-LDH (NiFe-LDH/rGO) nanosheets as support materials to synthesize stable Pt electrocatalysts. Ultrafine Pt nanocrystals were directly grown on the NiFe-LDH/rGO nanosheets via photo-assisted *in situ* reduction of the adsorbed PtCl_4_2^−^ precursor solution. The as-prepared Pt_*x*_/NiFe-LDH/rGO electrocatalysts present significantly enhanced durability compared with that of their Pt_*y*_/Ni(OH)_2_/rGO counterparts for MOR. First-principle calculations reveal that the enhanced durability of Pt/NiFe-LDH/rGO electrocatalysts originates from the optimized interfacial contact between Pt NPs and site-specific NiFe-LDH support, in which the Fe^3+^-O(H)-Pt bonds can more efficiently immobilize Pt NPs than the Ni^2+^-O(H)-Pt bonds on the Ni(OH)_2_ support.

## Result and Discussion

### Strategy for synthesizing Pt/NiFe-LDH/rGO nanocomposites

Pt/NiFe-LDH/rGO electrocatalysts were synthesized by two steps. Primarily, negatively charged GO surface (ζ = −44.3 mV, Figure S1) was used as the template to adsorb Ni^2+^ and Fe^3+^ cations; urea was used as a precursor to provide a slow-, sustained-release source of hydroxyl^21^. Under hydrothermal reaction, GO can be reduced to rGO, and urea will decompose into hydroxyl, ammonia, and carbon dioxide (Equation 1). The hydroxyl formation provides an alkaline environment, which directly promotes the nucleation and growth of 2D NiFe-LDH on rGO (Equation 2)^22^.

1 $${\text{CO}(\text{NH}}_{2}{)}_{2}+{{\rm{H}}}_{2}{\rm{O}}\to {{\rm{2NH}}}_{3}^{+}+{{\rm{OH}}}^{-}+{{\rm{CO}}}_{2}$$

2 $$\text{GO} \mbox{-} {({{\rm{Ni}}}^{2+})}_{{\rm{a}}{\rm{d}}}+\text{GO}-{({{\rm{Fe}}}^{3+})}_{{\rm{a}}{\rm{d}}}+{{\rm{OH}}}^{-}\to \text{NiFe} \mbox{-} \text{LDH}/{\rm{rGO}}$$

**Figure 1:**
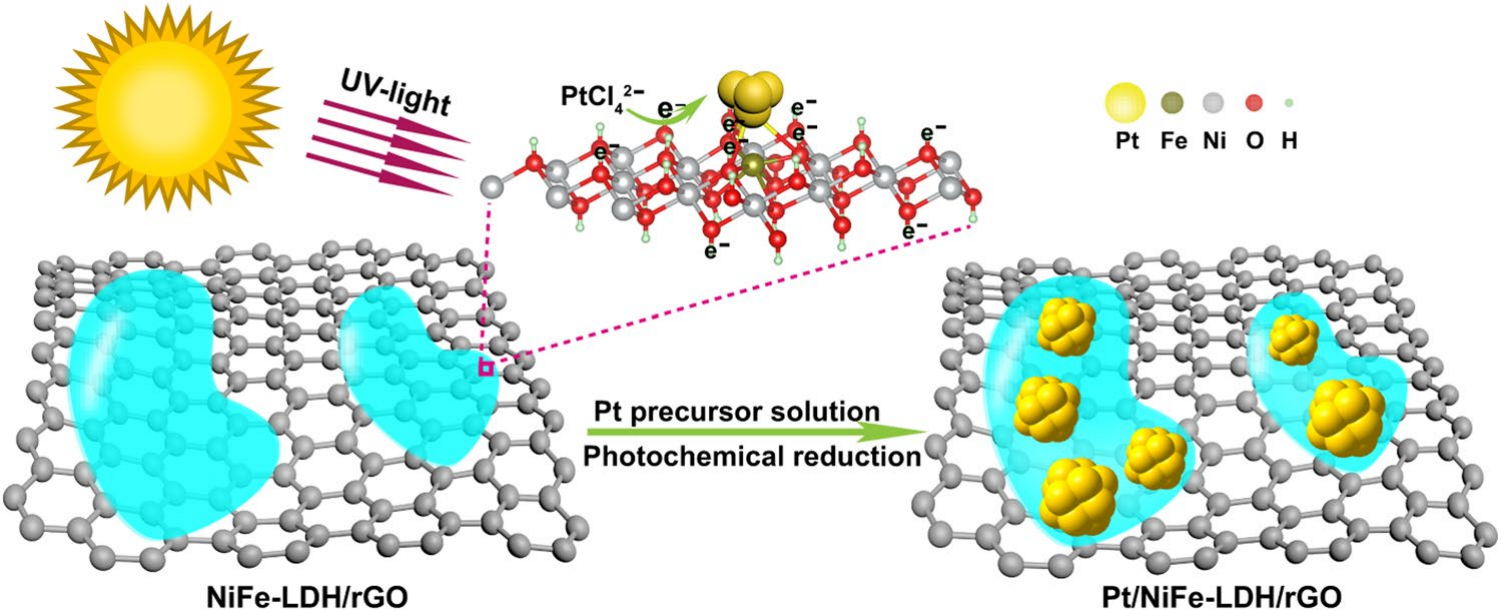
Schematic for the formation of graphene-supported platinum/nickel–iron layered double hydroxide (Pt/NiFe-LDH/rGO) nanocomposites.

The as-prepared NiFe-LDH/rGO and Ni(OH)_2_/rGO nanosheets were washed with deionized water and ethanol for several times. The zeta potentials of NiFe-LDH/rGO and Ni(OH)_2_/rGO nanosheets were +9.91 and −17.4 mV, respectively (Figure S2). Notably, given the positively charged surface originating from the unsaturated Fe^3+^O sites, the NiFe-LDH/rGO nanosheets showed better PtCl_4_^2−^ adsorption capacity than that of Ni(OH)_2_/rGO (Figure S3). Subsequently, as illustrated in Fig. 1, NiFe-LDH/rGO-supported Pt NPs were synthesized through photoassisted *in situ* reduction of adsorbed PtCl_4_^2−^. The growth of Pt NPs on NiFe-LDH can be expressed by the following formulas:

- Adsorption of PtCl_4_^2−^ on the surface of NiFe-LDH
  3 $$\text{NiFe} \mbox{-} \text{LDH}+{{\rm{PtCl}}}_{4}^{2-}\to \text{NiFe} \mbox{-} \text{LDH}-{({{\rm{PtCl}}}_{4}^{2-})}_{{\rm{ad}}}$$
- Creation of photogenerated electron–hole pairs
  4 $$\text{NiFe} \mbox{-} \text{LDH}+{hv}\to {e}^{-}+{h}^{+}$$
- Growth of Pt NPs on NiFe–LDH support

5 $$\text{NiFe} \mbox{-} \text{LDH} \mbox{-} ({{\rm{PtCl}}}_{4}^{2-}){\rm{ad}}+{{\rm{ne}}}^{-}\to {\rm{PtNPs}}/\text{NiFe} \mbox{-} \text{LDH}+{{\rm{nCl}}}^{-}$$

The ICP-AES result showed that the Ni^2+^ concentration in NiFe-LDH/rGO (79.125 μg mL^−1^) was close to that in Ni(OH)_2_/rGO (70.425 μg mL^−1^). In addition, a small amount of Fe^3+^ replaced Ni^2+^ in the Ni(OH)_2_ lattice in the preparation of NiFe-LDH/rGO (atomic ratio, Ni:Fe = 11.87:1). Notably, under the same Pt precursor addition, the loading amount of Pt NPs on NiFe-LDH/rGO was two times higher than that on Ni(OH)_2_/rGO after a trace amount of Fe^3+^ was introduced into the Ni(OH)_2_ lattice (Table S1). Two main factors are responsible for this result. First, the positively charged NiFe-LDH/rGO nanosheet surface possessed stronger PtCl_4_^2−^ adsorption capacity than that of Ni(OH)_2_/rGO nanosheets. This result indicated that a larger amount of adsorbed PtCl_4_^2−^ on NiFe-LDH/rGO nanosheets can participate in the photoreduction process than that on the Ni(OH)_2_/rGO supports. Second, the highly dispersed FeO_6_ octahedra in the LDHs matrix can hinder electron–hole recombination and promote the transfer of light-generated electrons to adsorbed reactants^23,24^. Therefore, more PtCl_4_^2−^ was reduced to Pt NPs on NiFe-LDH/rGO than that on Ni(OH)_2_/rGO.

### Characterization of Pt/NiFe-LDH/rGO nanocomposites

**Figure 2:**
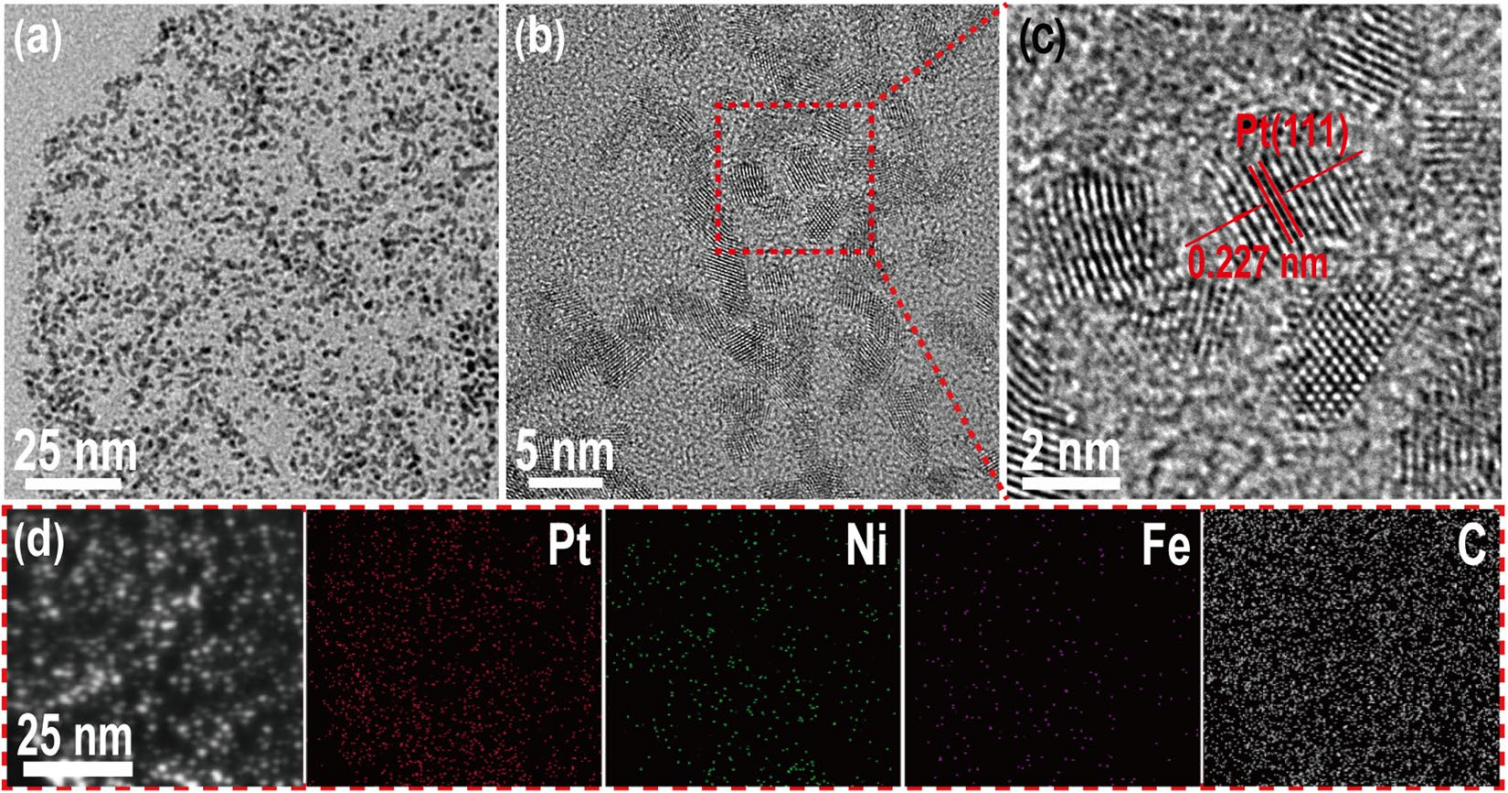
Representative (**a–b**) transmission electron microscopy (TEM), (**c**) HRTEM, (**d**) high-angle annular dark-field scanning TEM, and corresponding energy-dispersive spectroscopy elemental mapping images of Pt_0.54_/NiFe-LDH/rGO nanocomposite.

Figure 2a shows the representative TEM image of Pt_0.54_/NiFe-LDH/rGO nanocomposite. NiFe-LDH closely grew on the rGO nanosheets, and Pt nanocrystals with an average diameter or width of 2 nm were loaded on the surface of NiFe-LDH (Fig. 2b). Moreover, Pt nanocrystals displayed clear lattice fringes with an interplanar distance of 0.227 nm (Fig. 2c), which was in good agreement with the Pt (111) crystallographic plane. The corresponding HAADF image and EDS elemental mapping images (Fig. 2d) of the Pt_0.54_/NiFe-LDH/rGO nanocomposite indicated that Pt nanocrystals uniformly dispersed on NiFe-LDH/rGO without agglomeration and Fe atoms homogeneously dispersed in the Ni(OH)_2_ matrix. C signals covered the whole vision of the image index to the rGO, thereby demonstrating that rGO acted as a template for NiFe-LDH growth. As a comparison sample, Pt_0.12_/Ni(OH)_2_/rGO (Figure S4**)** showed similar morphology with Pt_0.54_/NiFe-LDH/rGO and a good dispersity of Pt NPs on Ni(OH)_2_/rGO.

**Figure 3:**
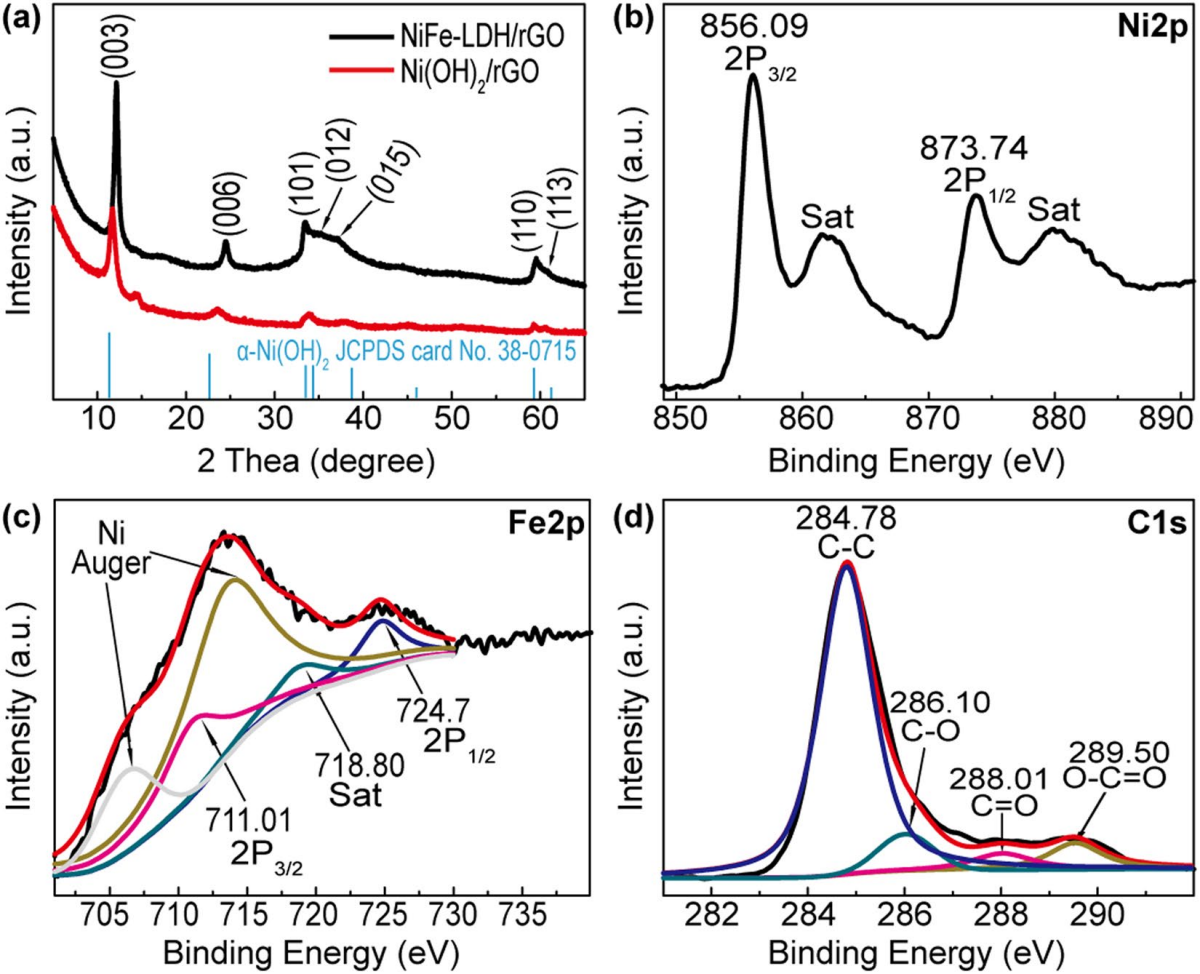
X-ray diffraction patterns (**a**) of NiFe-LDH/rGO and Ni(OH)_2_/rGO. The high-resolution X-ray photoelectron spectroscopy spectra of NiFe-LDH/rGO in (**b**) Ni 2p, (**c**) Fe 2p, and (**d**) C 1 s regions.

All diffraction peaks shown in the XRD patterns (Fig. 3a) of both NiFe-LDH/rGO and Ni(OH)_2_/rGO nanosheets matched well with the α-phase Ni(OH)_2_ (JCPDS No. 38-0715). The high–resolution XPS spectrum of Ni 2p in the NiFe-LDH/rGO (Fig. 3b) displayed two main peaks located at 856.09 and 873.74 eV, which suggested the +2 oxidation states of Ni^25^. In theory, Fe^3+^ is preferable than Fe^2+^ in fabricating NiFe-LDH because its ionic radius is similar to that of Ni^2+ ^^26^. As shown in Fig. 3c, the Fe 2p_3/2_ peaks overlapped with the Ni Auger peaks (near 706 and 712 eV). Thus, the satellite peak (718.80 eV) near the Fe 2p_3/2_ peak was selected as an indicator of the Fe^3+^ valence state^16,27^. The distances between the satellite peak (718.80 eV) and Fe 2p_1/2_ (724.70 eV) and Fe 2p_3/2_ (711.01 eV) were 5.90 and 7.79 eV, respectively. Quantitative analysis proved the +3 oxidation state of Fe^28^. In Fig. 3d, three peaks located at 284.78, 286.10, and 288.01 eV corresponded to C-C, C-O, and C=O bonds, respectively. The C-O and C=O peaks were evidently weaker than those of GO, thereby suggesting that GO was reduced after the hydrothermal reaction (Figure S5)^29^. Additionally, the emerging peak at 289.50 eV originated from the intercalated carbonate molecule (CO_3_^2−^) in the NiFe-LDH nanosheets^30^. For comparison, the chemical compositions of the Ni(OH)_2_/rGO were also corroborated by XPS, and results are summarized in Figure S6.

### Electrochemical performance of catalysts

**Figure 4:**
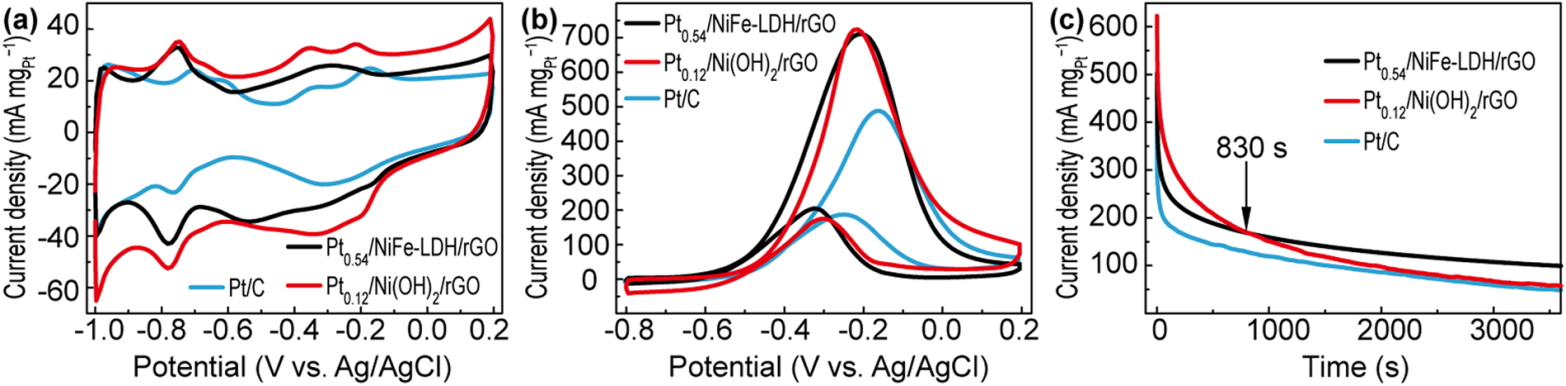
Cyclic voltammetry (CV) curves of Pt_0.54_/NiFe-LDH/rGO, Pt_0.12_/Ni(OH)_2_/rGO and commercial Pt-C (**a**) in N_2_-saturated KOH (1 M) at a scan rate of 50 mV s^−1^ and (**b**) in KOH (1 M)/CH_3_OH (1 M) at a scan rate of 50 mV s^−1^. (**c**) Chronoamperometry (CA) curves of Pt_0.54_/NiFe-LDH/rGO, Pt_0.12_/Ni(OH)_2_/rGO and commercial Pt-C in KOH (1 M) + CH_3_OH (1 M).

**Figure 5:**
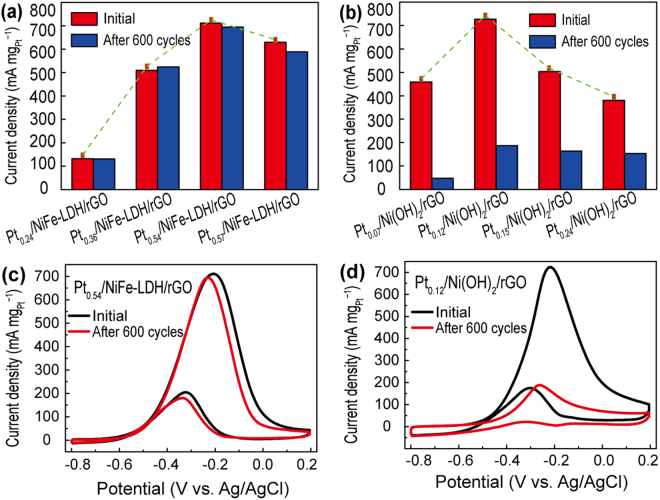
Peak currents of (**a**) Pt_*x*_/NiFe-LDH/rGO (*x* = 0.24, 0.36, 0.54, and 0.57) and (**b**) Pt_*y*_/Ni(OH)_2_/rGO (*y* = 0.07, 0.12, 0.15, and 0.24) at different cycle numbers. CV curves of (**c**) Pt_0.54_/NiFe-LDH/rGO and (**d**) Pt_0.12_/Ni(OH)_2_/rGO at different cycle numbers (1 M KOH +1 M CH_3_OH, scan rate: 50 mV s^−1^).

Figure 4a illustrates the typical CV curves of Pt_0.54_/NiFe-LDH/rGO, Pt_0.12_/Ni(OH)_2_/rGO and commercial Pt/C. On the basis of integrated hydrogen desorption region^31^, the calculated result showed that the ECSA values of Pt catalysts on Pt_0.54_/NiFe-LDH/rGO (609.01 cm^2^ mg^−1^) and Pt_0.12_/Ni(OH)_2_/rGO (767.62 cm^2^ mg^−1^) were higher than that of commercial Pt/C (498.86 cm^2^ mg^−1^). And given that the comparatively low loading amount and higher dispersity of Pt NPs, as verified by the ICP-AES analysis, Pt_0.12_/Ni(OH)_2_/rGO (Pt concentration, 28.53 µg mL^−1^) ternary hybrids showed slightly higher electrocatalytic activity than that of Pt_0.54_/NiFe-LDH/rGO (Pt concentration, 146.80 µg mL^−1^) during MOR. Also, both two hydroxides supported Pt catalysts displayed higher peak current than the commercial Pt/C electrocatalysts (Fig. 4b). Figure 4c presents the CA curves of Pt_0.54_/NiFe-LDH/rGO, Pt_0.12_/Ni(OH)_2_/rGO and Pt/C at the potential of −0.2 V for 3600 s. This potential corresponded to the methanol oxidation peaks during forward sweep. The current density of Pt_0.54_/NiFe-LDH/rGO surpassed Pt_0.12_/Ni(OH)_2_/rGO catalyst after 830 s, and then showed the highest current density during the prolonged CA test. These results illustrated that the Pt_0.54_/NiFe-LDH/rGO electrocatalysts may possess better stability than that of its Pt_0.12_/Ni(OH)_2_/rGO and Pt/C counterparts. To evaluate the overall durability of Pt_*x*_/NiFe-LDH/rGO and Pt_*y*_/Ni(OH)_2_/rGO electrocatalysts during MOR testing and figure out which part (Ni^2+^O_6_ or Fe^3+^O_6_ octahedra in hydroxides supports) is decisive in immobilizing Pt NPs, long-term CV tests were applied at a potential ranging from −0.8 V to 0.2 V at a scan rate of 50 mV s^−1^. According to Fig. 5a and b, both types of electrocatalysts presented a volcanic-type trend. The mass activity of Pt_*x*_/NiFe-LDH/rGO and Pt_*y*_/Ni(OH)_2_/rGO increased with the increasing amount of loaded Pt initially but decreased when excessive amount of Pt precursor was reduced on the hydroxide support (Figures S7 and S8). Notably, after 600 cycles of CV test in methanol and potassium hydroxide mixture, each Pt_*x*_/NiFe-LDH/rGO electrocatalyst can maintain more than 93% of their catalytic activity. However, all the Pt_*y*_/Ni(OH)_2_/rGO electrocatalysts showed a considerable loss of catalytic activity during MOR, and less than 40% initial catalytic activity of Pt_*y*_/Ni(OH)_2_/rGO can be retained after 600 cycles of CV tests. Among these samples, Pt_0.54_/NiFe-LDH/rGO and Pt_0.12_/Ni(OH)_2_/rGO presented the optimal peak current density for Pt_*x*_/NiFe-LDH/rGO and Pt_*y*_/Ni(OH)_2_/rGO, respectively. In particular, the loading amount of Pt on Pt_0.54_/NiFe-LDH/rGO was 4.5 times higher than that on Pt_0.12_/Ni(OH)_2_/rGO. Commonly, the high loading amount of Pt was likely to cause the aggregation and deactivation of Pt^32,33^. Nevertheless, after 600 cycles of CV tests, 97.6% initial peak current density of the Pt_0.54_/NiFe-LDH/rGO electrocatalyst can be maintained (from 711.04 mA mg^−1^ to 694.28 mA mg^−1^), whereas the peak current density of Pt_0.12_/Ni(OH)_2_/rGO decreased from 724.28 mA mg^−1^ to 188.75 mA mg^−1^ (only 26.1% initial peak current density was retained) (Figs 5c,d and S9). Moreover, after 1200 cycles of CV tests, the peak current density of Pt_0.54_/NiFe-LDH/rGO still remained at 86.5% (Figure S10). By comparison, the commercial Pt/C electrocatalyst can only retain 61.3% of its initial peak current (Figure S11). Amazingly, after the introduction of Fe^3+^ into Ni(OH)_2_ lattice, the overall performance of Pt*x*/NiFe-LDH/rGO electrocatalysts were dramatically enhanced than Pt*y*/Ni(OH)_2_/rGO as well as other literature reported results (Table S2). We ascribed such encouraging result, especially the durability of Pt_*x*_/NiFe-LDH/rGO, to the effect of ferric sites in Ni(OH)_2_ lattice.

To further understand the morphology and structure change of Pt_*x*_/NiFe-LDH/rGO and Pt_*y*_/Ni(OH)_2_/rGO after MOR cycling tests, systematical TEM characterization and size distribution of Pt NPs were carried out to study the change in the size of as-obtained electrocatalysts before and after the long-term MOR tests. Prior to MOR tests, both the Pt NPs loaded on NiFe-LDH/rGO and Ni(OH)_2_/rGO gradually grew with a quantitatively increasing addition of Pt precursors (Figure S12 and S13). Differently, the Pt NPs loaded on NiFe-LDH/rGO attached to one another and gradually grew into worm-like structures due to the high loading amount of Pt. By contrast, the Pt NPs loaded on Ni(OH)_2_/rGO always maintained a granular morphology. For consistency, the width of worm-like Pt and the diameter of particle-like Pt presented on NiFe-LDH/rGO were counted together when the size distribution of Pt nanocrystals was surveyed.

**Figure 6:**
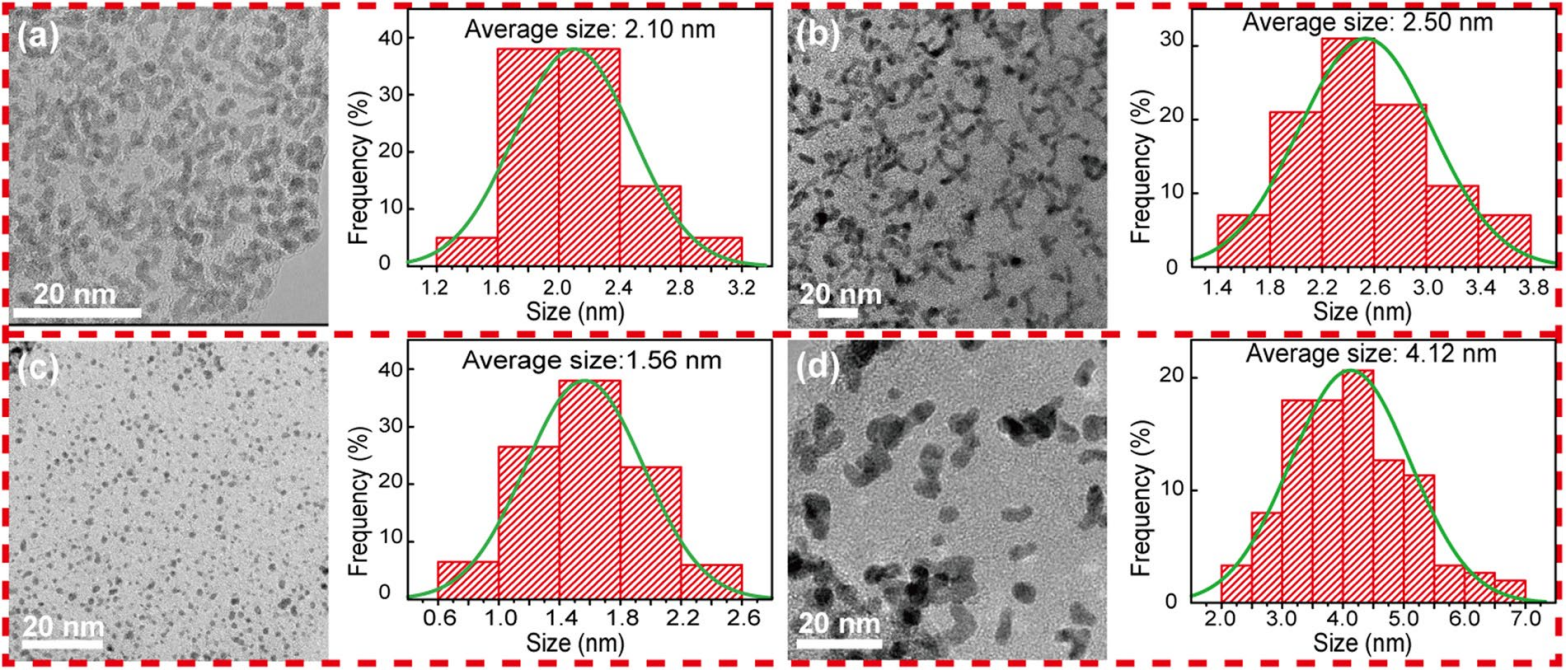
TEM image and Pt nanoparticle (NP) size distribution histogram of (**a**) initial Pt_0.54_/NiFe-LDH/rGO, (**b**) Pt_0.54_/NiFe-LDH/rGO after 600 cycles of CV test, (**c**) initial Pt_0.12_/Ni(OH)_2_/rGO, and (**d**) Pt_0.12_/Ni(OH)_2_/rGO after 600 cycles of CV test. CV tests were performed in1 M KOH+ 1 M CH_3_OH at a scan rate of 50 mv s^−1^.

First, the most remarkable samples, namely, Pt_0.54_/NiFe-LDH/rGO and Pt_0.12_/Ni(OH)_2_/rGO, were selected to clarify the variety in the size of Pt NPs before and after 600 cycles of CV test. As shown in Fig. 6, the average size of Pt NPs in Pt_0.12_/Ni(OH)_2_/rGO increased from 1.56 nm to 4.12 nm. The Pt nanocrystal in Pt_0.54_/NiFe-LDH/rGO showed a slight increase from 2.1 nm to 2.5 nm in average size. In general, the surface energy of Pt NPs is closely linked to its size, and the initial Pt NPs of two contrasting samples should be similar in size^34^. Therefore, Pt_0.36_/NiFe-LDH/rGO and Pt_0.24_/Ni(OH)_2_/rGO were selected as contrasting samples because of their same average size of Pt nanocrystals prior to MOR testing (1.89 nm vs. 1.84 nm). After 600 cycles of CV tests, as given in Figure S14, the average size of Pt NPs on Pt_0.24_/Ni(OH)_2_/rGO increased markedly to 4.46 nm. Pt_0.36_/NiFe-LDH/rGO showed only a 0.29 nm increment in mean size (from 1.89 nm to 2.18 nm). These results confirmed that NiFe-LDH support can efficiently anchor Pt NPs, whereas the Ni(OH)_2_ support cannot. Consequently, severe agglomeration of Pt NPs occurred on the Ni(OH)_2_ support during MOR, which caused the quick deactivation of Pt_*x*_/Ni(OH)_2_/rGO electrocatalysts.

In addition to the size effect, two main factors, namely, the instability of support and weak metal–support contact, are also closely linked to the agglomeration of Pt NPs on supports^35,36^. Relevant literature demonstrated that in an alkaline electrolyte, α-Ni(OH)_2_ is prone to convert into β-Ni(OH)_2_ during long-term electrochemical process^37^. The phase-transition-induced instability of support may result in the aggregation of Pt NPs, and this process can be detected by ongoing CV tests^38^. Thus, to evaluate the electrochemical stability of Ni(OH)_2_/rGO and NiFe-LDH/rGO supports, long-term CV tests were conducted in KOH solution (1 M), which contained methanol (1 M). Figure S15 shows that after 600 cycles of CV test, the latest CV curves of Ni(OH)_2_/rGO and NiFe-LDH/rGO supports exhibited almost no change compared with their initial ones. The favorable electrochemical stability of both supports is mainly due to the introduction of rGO^39^. The above results indicated that the aggregation of Pt NPs was not caused by the phase transition of hydroxides support.

**Figure 7:**
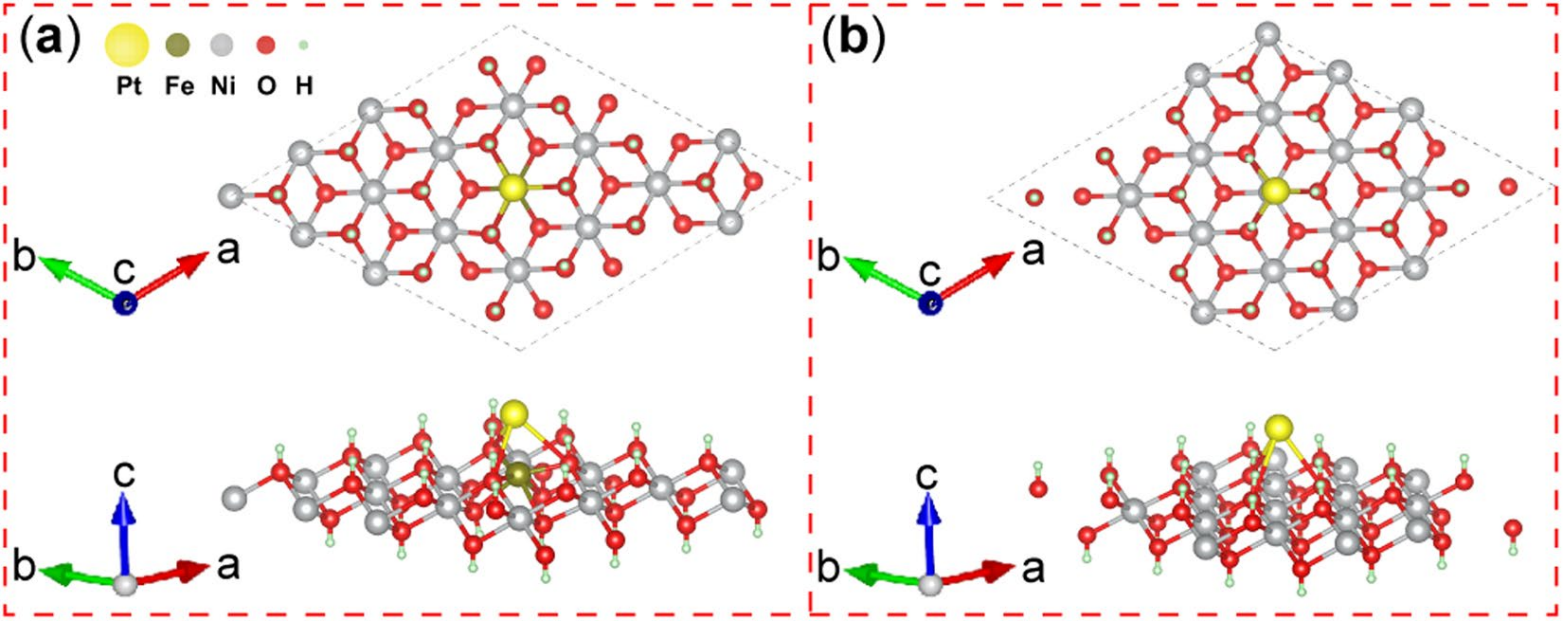
Atomic structure of Pt atom bonding to the (**a**) ferric site in NiFe-LDH and (**b**) nickel site in Ni(OH)_2_ nanosheets.

On the basis of above analysis, first-principle calculations were performed to analyze the contact interface between Pt NPs and supports (Fig. 7), in which the metal–support interface may be directly linked to the stability of Pt NPs^40^. The adsorption energy (*E*_ad_) of Pt atom adsorbed on the sheets is defined as *E*_ad_ = *E*_tot_ − *E*_sheet_ − *E*_Pt_, where *E*_tot_ is the total energy of system with sheets and adsorbed Pt atoms, *E*_sheet_ is the energy of the optimized pure sheets, and *E*_Pt_ is the energy of one Pt atom in the vacuum. Therefore, a remarkably negative *E*_ad_ value indicated strong the interaction of Pt atoms with Ni(OH)_2_ sheets or NiFe-LDH sheets. The adsorption energy *E*_ad_ of Pt atom adsorbed on the NiFe-LDH sheets (via the Fe^3+^-O(H)-Pt bonding) was −2.87 eV, which was lower than that of Pt atom adsorbed on Ni(OH)_2_ sheets (via the Fe^3+^-O(H)-Pt bonding, −2.08 eV). The theoretical calculation revealed that the interaction of Pt atom with site-specific NiFe-LDH sheets was markedly stronger than that of Pt atom with Ni(OH)_2_ sheets. Thus, Pt/NiFe-LDH/rGO electrocatalysts were markedly more stable than Pt/Ni(OH)_2_/rGO electrocatalysts.

## Conclusion

This work synthesized 2D NiFe-LDH/rGO and Ni(OH)_2_/rGO supports via a graphene-templated and urea-assisted hydrothermal route. Afterward, ultrafine Pt nanocrystals were loaded on NiFe-LDH/rGO and Ni(OH)_2_/rGO supports through photo-assisted *in situ* reduction of adsorbed PtCl_4_^2−^. Both ternary composites displayed ultrafine Pt nanocrystals anchored on graphene-supported hydroxide nanosheets. Long-term MOR tests showed that after the introduction of ferric sites in Ni(OH)_2_ lattice, the Pt/NiFe-LDH/rGO nanocatalysts displayed dramatically enhanced durability compared with that of Pt/Ni(OH)_2_/rGO as well as commercial Pt/C electrocatalysts. The systematical TEM characterization and size distribution of Pt NPs of as-obtained electrocatalysts before and after long-term MOR tests, together with the chemical stability tests of NiFe-LDH/rGO and Ni(OH)_2_/rGO supports, confirmed that Fe^3+^ partially replaced Ni^2+^ in Ni(OH)_2_ lattice can efficiently enhance the stability of supported Pt NPs. First-principle calculations of the resultant M-O(H)-Pt (M = Fe^3+^, Ni^2+^) interfacial structure further corroborated that the NiFe-LDH nanosheets can provide stronger bonding sites (via the Fe^3+^-O(H)-Pt bonds) to immobilize Pt NPs than that of Ni(OH)_2_ nanosheets (via the Ni^2+^-O(H)-Pt bonds). Our results suggested that the excellent stability of Pt/NiFe-LDH/rGO was attributed to the strong contact between Pt NPs and site-specific NiFe-LDH support. In a broad sense, the strong contact between Pt NPs and site-specific supports elucidated in this work may provide a general strategy to synthesize site-specific anchoring Pt electrocatalysts with long-term stability and high catalytic activity.

## Experimental Section

### Chemical reagents and materials

All reagents used in the present experiments were of analytical grade and applied without further purification. Graphite powder was purchased from Tianjin Guangfu Fine Chemical Research Institute. GO was prepared from graphite with a modified Hummers method^41^. Commercial 20% Pt/C was purchased from Alfa Aesar. Deionized water (resistance, 18 MΩ cm^−1^) was used throughout all experiments.

### Synthesis of NiFe-LDH/rGO and Ni(OH)_2_/rGO

The 2D NiFe-LDH/rGO hybrids were synthesized with a graphene-templated, urea-assisted hydrothermal method. Briefly, 30 mg of Ni(NO_3_)_2_·6H_2_O, 1.07 mg of Fe(NO_3_)_3_·9H_2_O, and 120 mg of urea were subsequently dissolved in 15 mL of GO aqueous solution (0.25 g L^−1^) under ultrasonic conditions. The obtained uniform solution was transferred into a Teflon cup in a stainless steel-lined autoclave. The autoclave was maintained at 100 °C for 12 h and cooled to room temperature naturally. The final products were washed with deionized water for several times and redispersed in 15 mL of aqueous solution. The 2D Ni(OH)_2_/rGO composite material was prepared using the same procedure but without the addition of ferric salt.

### Synthesis of Pt_*x*_/NiFe-LDH/rGO and Pt_*y*_/Ni(OH)_2_/rGO

The 2D NiFe-LDH/rGOs or Ni(OH)_2_/rGO-supported Pt NPs were prepared via photoreduction of the Pt precursor solution. Prior to illumination, 2 mL of as-prepared NiFe-LDH/rGO or Ni(OH)_2_/rGO hybrids and a certain amount of sodium chloroplatinate solution (Na_2_PtCl_4_·4H_2_O, 4 mg mL^−1^) were added into a 50 mL quartz tube containing 30 mL of deionized water. After ultrasonic treatment for 30 min, the mixtures were stirred intensely and illuminated by a 300 W mercury lamp for 90 min with a distance of 10 cm at room temperature. The black precipitates were collected by centrifugation and washed with deionized water for five times. The final products were redispersed in 4 mL of ionized water for electrochemical measurements. A series of electrocatalysts was obtained and labeled as Pt_*x*_/NiFe-LDH/rGO (*x* = 0.17, 0.24, 0.36, 0.54, 0.57) and Pt_*y*_/Ni(OH)_2_/rGO (*y* = 0.07, 0.12, 0.15, 0.24) by adjusting the amounts of added Na_2_PtCl_4_·4H_2_O solution (0.5, 1.0, 1.5, 2.0, and 2.5 mL) and without changing any other parameter. The *x* or *y* values were obtained by the molar ratios of Pt and M (M = Ni or Fe). The mass concentrations of Ni, Fe, and Pt in different prepared catalysts were determined by inductively coupled plasma atomic emission spectroscopy (ICP-AES) analysis.

### Characterization

The phase structure of the prepared catalysts was analyzed through X-ray diffraction (XRD) with a Philips X’Pert system with Cu Ka radiation (*λ* = 0.15419 nm). The surface chemical constituents of the prepared catalysts were analyzed by X-ray photoelectron spectroscopy (XPS, Thermo ESCACLB 250). The surface zeta potentials of GO, NiFe-LDH/rGO, and Ni(OH)_2_/rGO were measured by a Malvern instrument (Nano-zs90). Transmission electron microscopy (TEM) images, high-angle annular dark-field (HAADF) scanning TEM images, and energy-dispersive spectroscopy (EDS) elemental mapping images were captured by a FEI Tecnai TF20 operated at 200 kV.

### Electrochemical measurements

Prior to electrode preparation, the catalyst was ultrasonicated for 30 min to form a uniform suspension. A glassy carbon electrode (3 mm in diameter) was polished to a mirror finish with alpha alumina powder (0.05 μm) and ultrasonically cleaned in ethanol for 3 min. Subsequently, 5 μL of the as-prepared catalyst suspension was drop-casted onto a working electrode and dried at ambient temperature. When the slurry was dried, 10 μL of 0.05 wt.% Nafion solution was covered onto the dried sample. All electrochemical experiments were examined at room temperature (25 ± 1 °C). A standard three-electrode electrochemical workstation (Zahner IM6ex) was used for all electrochemical experiments. A Pt wire and an Ag/AgCl electrode were adopted as the counter and reference electrodes, respectively. All potentials in this study were reported with respect to Ag/AgCl electrode. The weight of Pt on each working electrode was calculated through ICP-AES tests. Prior to any electrochemical measurement, electrocatalysts were preactivated in N_2_-saturated KOH aqueous solution (1 M) by cyclic voltammetry (CV) cycling at the potential ranging from −1.0 V to 0.2 V at 100 mV s^−1^ for 30 cycles until the curve stabilized. Afterward, the CV curves were collected at a slow scan rate of 50 mV s^−1^ to calculate the electrochemically active surface area (ECSA) of different electrocatalysts. To evaluate the methanol oxidation performance, both the CV (from −0.8 V to 0.2 V, 50 mV s^−1^) and chronoamperometry (CA, 3600 s) tests were performed in a hybrid solution of KOH (1 M) containing CH_3_OH (1 M). Long-term CV cycling tests were also applied to assess the stability of each catalyst.

### Theoretical calculation

First-principle calculations were conducted to evaluate the adsorption energy of Pt on the pure Ni(OH)_2_ sheets and NiFe-LDH sheets. Structure relaxation and total energy calculations were carried out using density functional theory within the generalized gradient approximation, as instructed in the VASP 5.4 package^42^. Electronic exchange and correlation are described by Perdew-Burke-Ernzerhof function^43^. All electron plane-wave basis sets with the projector augmented wave potentials were adopted with 2s^2^2p4, 3d^8^4s2, and 3d^7^4s1, which were treated as valence electron configuration for O, Ni, and Fe, respectively. The cutoff energy was 500 eV. A sufficiently dense k-point sampling was evaluated with energy tolerance in 1 meV/atom. A vacuum higher than 15 Å thick was inserted in each model to avoid interaction with imaging free sheets. The ground state geometries were obtained by minimizing the forces on each atom to less than 0.01 eV/Å. The determined magnetic configuration of Ni(OH)_2_ sheets were antiferromagnetic.

## Electronic supplementary material

Supporting information
