# Supplementary material for: Strong Fe3+-O(H)-Pt Interfacial Interaction Induced Excellent Stability of Pt/NiFe-LDH/rGO Electrocatalysts
Source: Sci Rep. 2018 Jan 22;8:1359. doi: 10.1038/s41598-018-19876-z (PMC5778055; doi:10.1038/s41598-018-19876-z)
Supplement: Supplementary file 1 — Supporting information [file 41598_2018_19876_MOESM1_ESM.pdf]

Supporting Information

Strong Fe^{3+}-O(H)-Pt Interfacial Interaction Induced Excellent Stability of Pt-NiFe-LDH/rGO Electrocatalysts

Yechuang Han [a, b‡], Pengfei Li [a‡], Jun Liu [a*], Shouliang Wu [a], Yixing Ye [a], Zhenfei Tian [a] and Changhao Liang [a, b*]

[a]Key Laboratory of Materials Physics and Anhui Key Laboratory of Nanomaterials and Nanotechnology, Institute of Solid State Physics, Hefei Institutes of Physical Science, Chinese Academy of Sciences, Hefei 230031, China.

[b]Department of Materials Science and Engineering, University of Science and Technology of China, Hefei 230026, China.

‡These authors contributed equally to this work.

***Corresponding author**

E-mail: jliu@issp.ac.cn; chliang@issp.ac.cn

Tel: +86 55165591320; Fax: +86 55165591434;

Figures S1 to S15

Tables S1 and S2

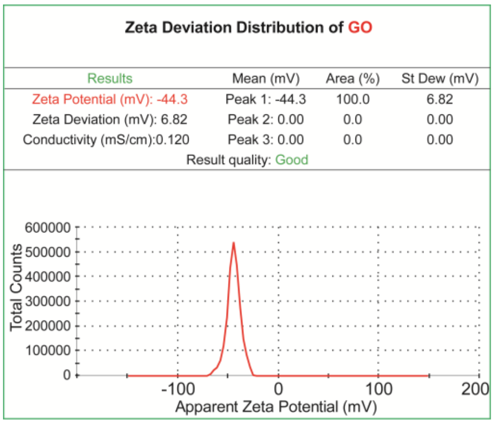

Figure S1 Zeta potential reports of GO.

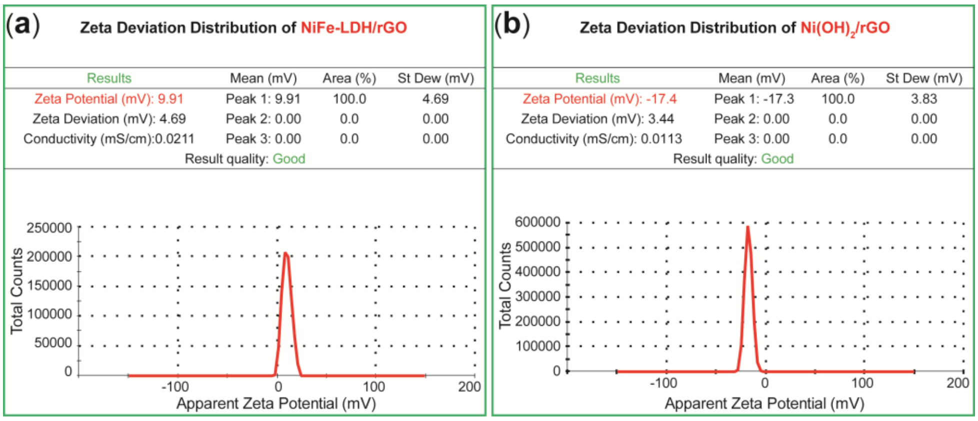

Figure S2 Zeta potential reports of (a) NiFe-LDH/rGO, (b) $Ni(OH)_2$/rGO. Before tests, both NiFe-LDH/rGO and $Ni(OH)_2$/rGO solution were diluted 4 times for better dispersion.

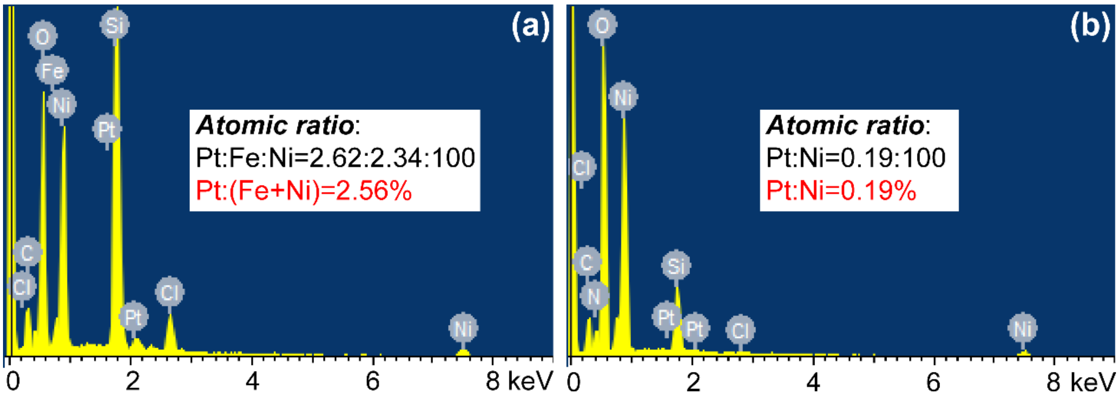

Figure S3 EDS spectra of (a) NiFe-LDH/rGO with adsorbed $PtCl_4^{2-}$, (b) $Ni(OH)_2$/rGO with adsorbed $PtCl_4^{2-}$. The $PtCl_4^{2-}$ adsorption test was carried out by the following procedure. Firstly, 0.5 ml rare NiFe-LDH/rGO and $Ni(OH)_2$/rGO solution were dispersed in 5 ml deionized water containing 0.5 ml $Na_2PtCl_4 \cdot 4H_2O$ (4 mg/mL), respectively. Then, the hybrid solution was agitated

for 30 min in the darkroom. The final products was ultrasonic cleaned with ethanol and water for 8 times to remove unabsorbed $PtCl_4^{2-}$, and then drop-cast onto a silicon wafer for EDS analyze.

Table S1 Comparison of the Pt loading amount on supports.

$Na_2PtCl_4 \cdot 4H_2O_{aq}$ addition (1 g/250 ml)	**Pt/NiFe-LDH/rGO** (Atomic ratio: m = Pt/(Ni+Fe))	**Pt/Ni(OH)$_2$/rGO** (Atomic ratio: n = Pt/Ni)	**Ratio of loaded Pt** (m/n)
0.5 ml	0.17	0.07	**2.43**
1.0 ml	0.24	0.12	**2.00**
1.5 ml	0.36	0.15	**2.40**
2.0 ml	0.54	0.24	**2.25**

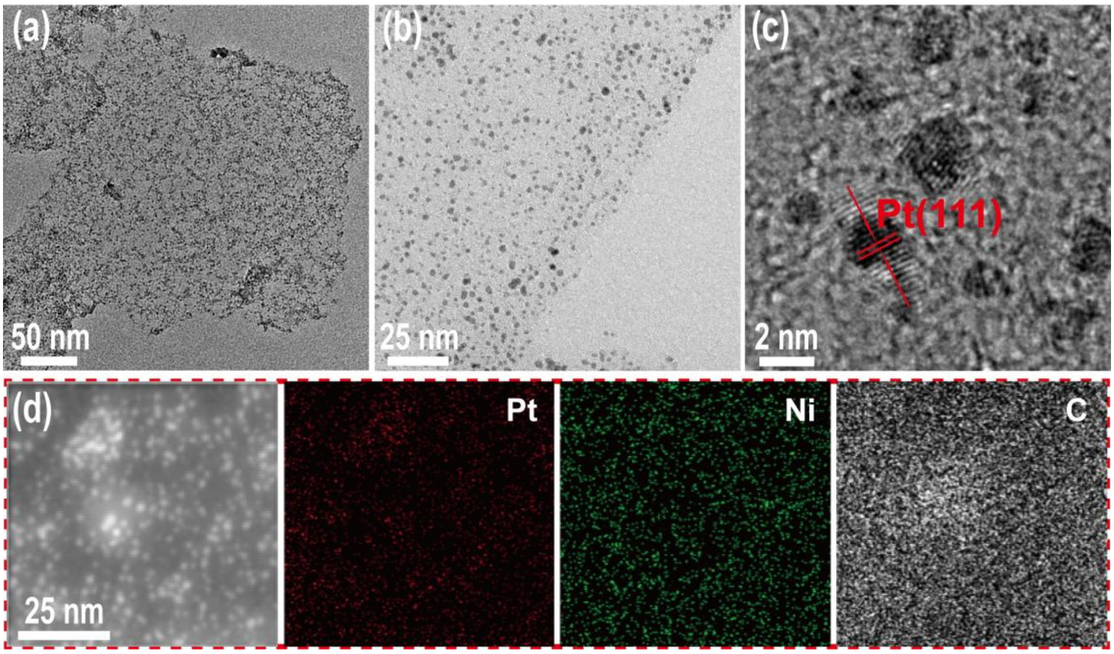

Figure S4 Representative (a-b) TEM, (c) HRTEM, (d) HAADF-STEM images and its corresponding EDS mapping of $Pt_{0.12}/Ni(OH)_2/rGO$ nanocatalysts.

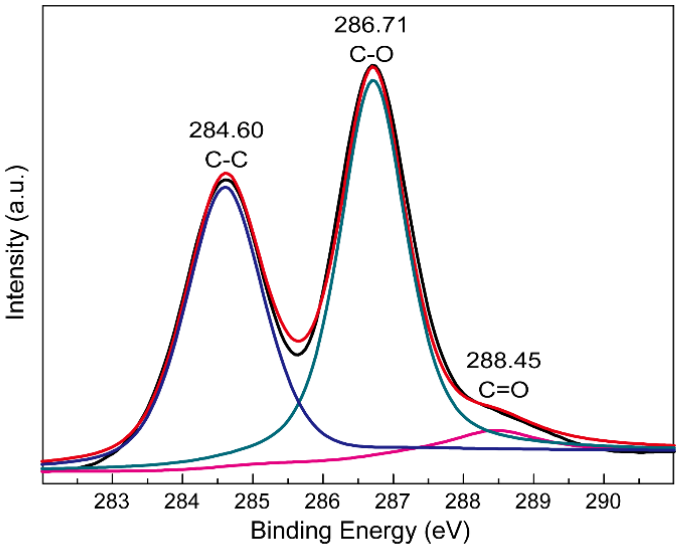

Figure S5. High-resolution C1s XPS spectra of GO.

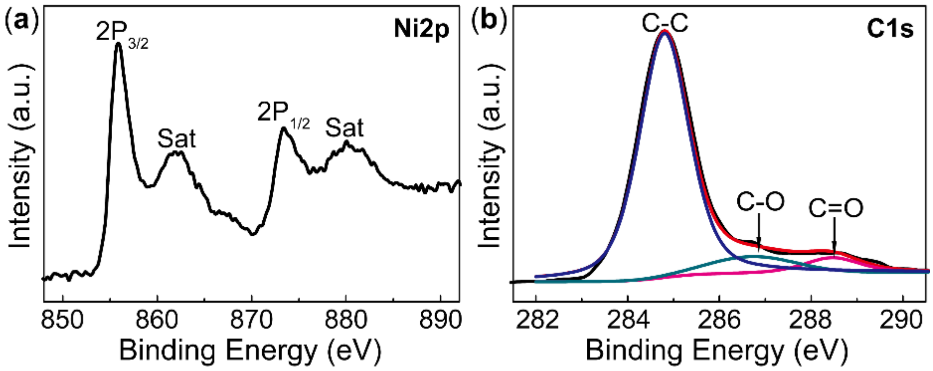

Figure S6. The high resolution XPS spectra of $Ni(OH)_2$/rGO in (a) Ni 2p region, (c) C 1s region.

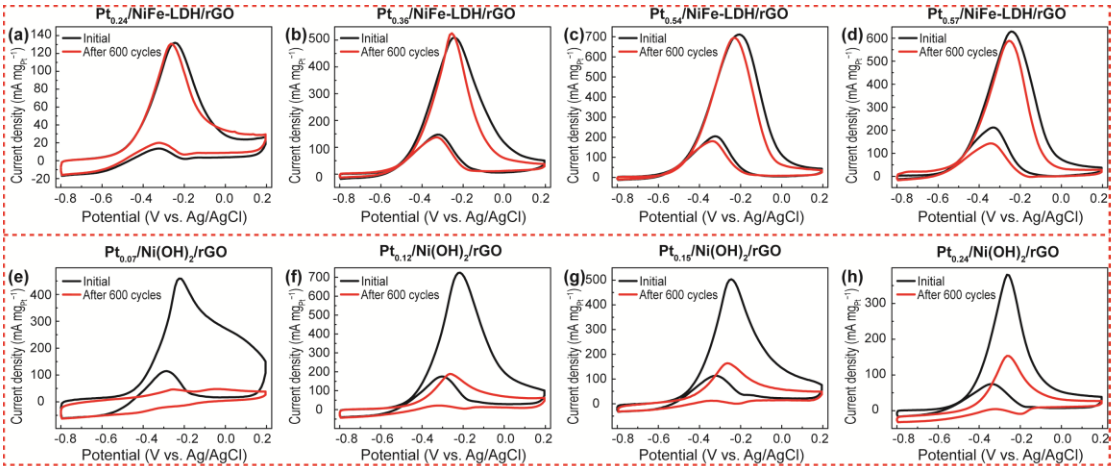

Figure S7. CV curves at different cycle numbers of prepared catalysts (a-d) Pt_x/NiFe-LDH/rGO (x=0.24, 0.36, 0.54 and 0.57) and (e-h) $Pt_y/Ni(OH)_2$/rGO (y=0.07, 0.12, 0.15 and 0.24) in 1M KOH+1M CH_3OH at a scan rate of 50mV s^{-1}.

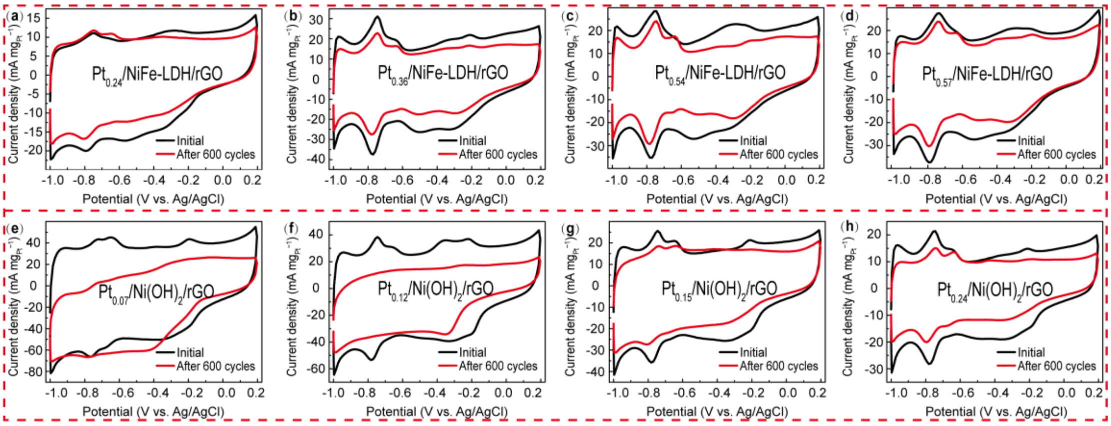

Figure S8. CV curves at different cycle numbers of prepared catalysts (a-d) Pt_x/NiFe-LDH/rGO (x=0.24, 0.36, 0.54 and 0.57) and (e-h) $Pt_y/Ni(OH)_2$/rGO (y=0.07, 0.12, 0.15 and 0.24) in 1M KOH at a scan rate of 50 mV s^{-1}.

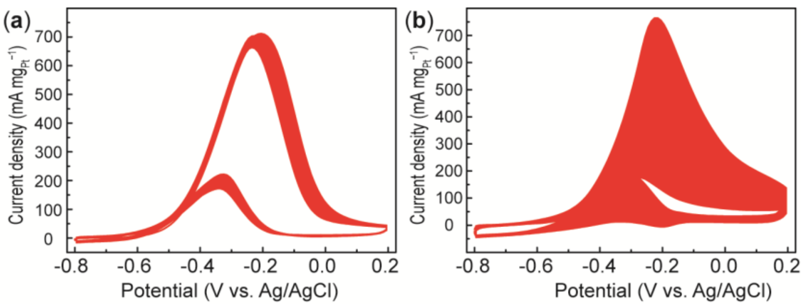

Figure S9. 600 cycles CV curves of prepared electrocatalysts (a) $Pt_{0.54}$/NiFe-LDH/rGO, (b) $Pt_{0.12}/Ni(OH)_2$/rGO in 1M KOH at scan rate of 50 mV s^{-1}.

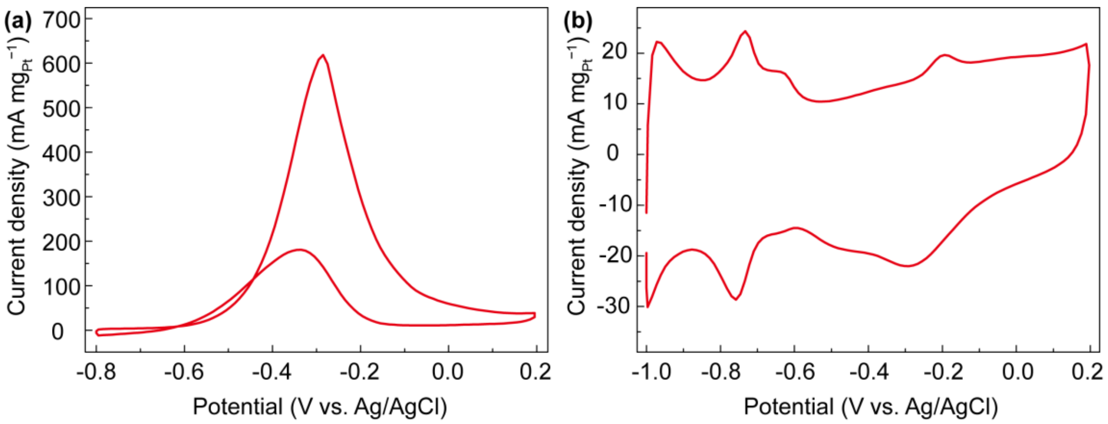

Figure S10. CV curves of $Pt_{0.54}$/NiFe-LDH/rGO in (a) 1M KOH+1M CH_3OH, (b) 1M KOH at a scan rate of 50 mV s^{-1} after 1200 cycles CV tests.

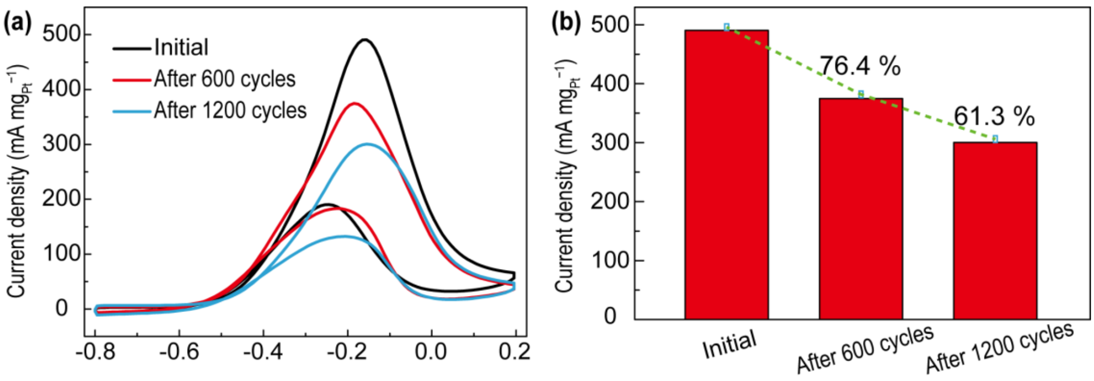

Figure S11. (a) CV curves of commercial 20% Pt/C at different cycle numbers in 1 M KOH + 1 M CH_3OH at a scan rate of 50 mV s^{-1}. (b) Peak currents of commercial 20% Pt/C at different cycle numbers in 1 M KOH + 1 M CH_3OH at a scan rate of 50 mV s^{-1}.

Table S2. A summary of MOR activity and stability of Pt-based catalysts in literature.

Catalysts	Electrolyte	Stability	Initial Activity	Reference
Pt/NiFe-LDH/rGO	1 M KOH + 1 M methanol	86.5% remain After 1200 cycles	711.04 mA mg^{-1} (scan rate, 50 mV s^{-1})	This work
Pt/$Ni(OH)_2$/rGO	1 M KOH + 1 M methanol	26.1%remain After 600 cycles	727.25 mA mg^{-1} (scan rate, 50 mV s^{-1})	This work
Commercial Pt/C	1 M KOH + 1 M methanol	61.27% remain After 1200 cycles	490.40 mA mg^{-1} (scan rate, 50 mV s^{-1})	This work
Pt/rGO	0.5 M KOH + 0.5 M methanol	77.9% remain After 1000 cycles	552.5 mA mg^{-1} (scan rate, 50 mV s^{-1})	[1]
Pt/NiAl-LDH/rGO	0.5 M KOH + 1 M methanol	~83% remain After 5h CA test	504 mA mg^{-1} (scan rate, 50 mV s^{-1})	[2]
Pt/SnO_2/C	0.1 M $HClO_4$ + 0.1 M ethanol	~70% remain After 1000 cycles	Bellow 200 mA mg^{-1} (scan rate, 20 mV s^{-1})	[3]
Pt/SnO_2	0.5 M H_2SO_4 + 0.5 M methanol	Not Given	230.3 mA mg^{-1} (scan rate, 50 mV s^{-1})	[4]
Ni@Pt NPs	1 M KOH + 1 M methanol	Not Given	300 mA mg^{-1} (scan rate, 50 mV s^{-1})	[5]
Pt/rGO/TiO_2	1 M KOH + 1 M methanol	Not Given	507.4 mA mg^{-1} (scan rate, 50 mV s^{-1})	[6]

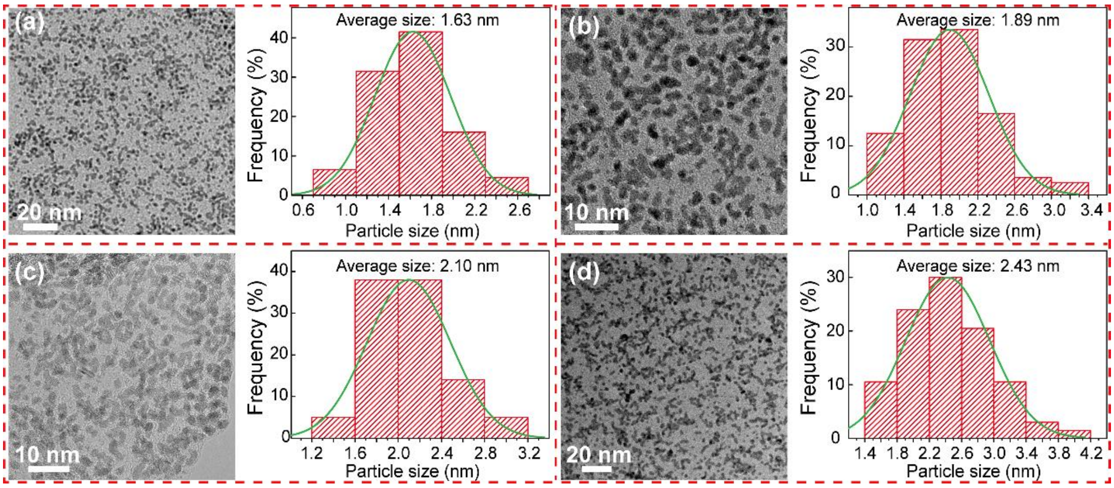

Figure S12. TEM imagine and Pt NPs size distribution histogram of (a) $Pt_{0.24}$/NiFe-LDH/rGO, (b) $Pt_{0.36}$/NiFe-LDH/rGO, (c) $Pt_{0.54}$/NiFe-LDH/rGO, (d) $Pt_{0.57}$/NiFe-LDH/rGO electrocatalysts.

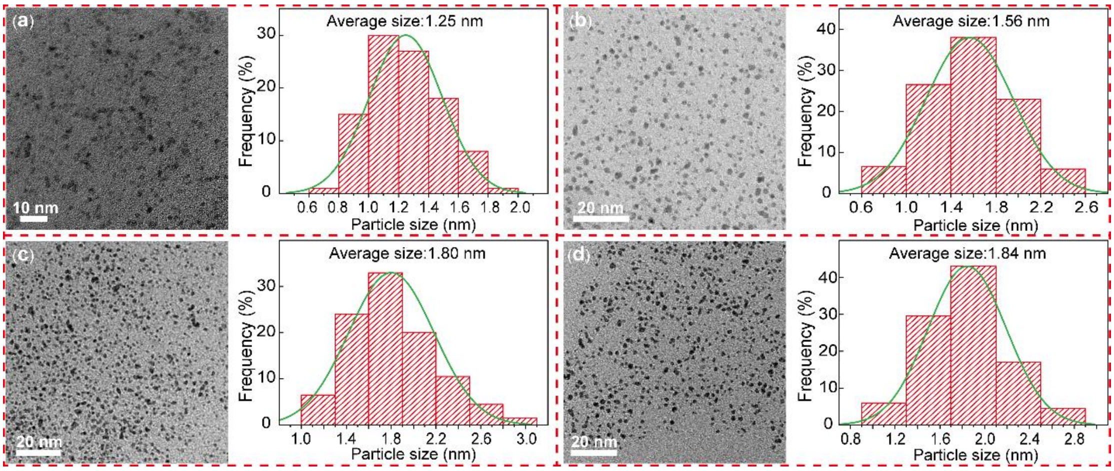

Figure S13. TEM imagine and Pt NPs size distribution histogram of (a) $Pt_{0.07}/Ni(OH)_2/rGO$, (b) $Pt_{0.12}/Ni(OH)_2/rGO$, (c) $Pt_{0.15}/Ni(OH)_2/rGO$, (d) $Pt_{0.24}/Ni(OH)_2/rGO$ electrocatalysts.

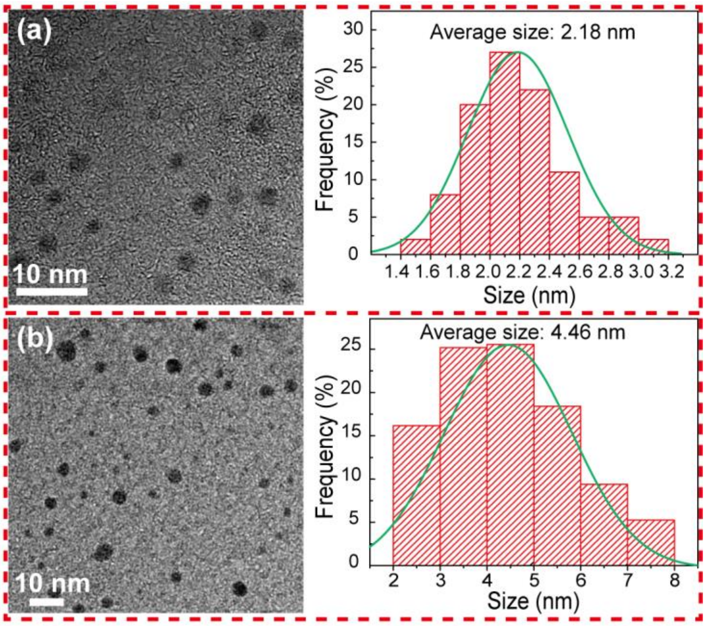

Figure S14.TEM imagine and Pt NPs size distribution histogram of (a) $Pt_{0.36}$/NiFe-LDH/rGO, (b) $Pt_{0.24}/Ni(OH)_2/rGO$ after 600 cycles CV test. CV tests, in 1M KOH+1M CH_3OH at a scan rate of 50 mv/s.

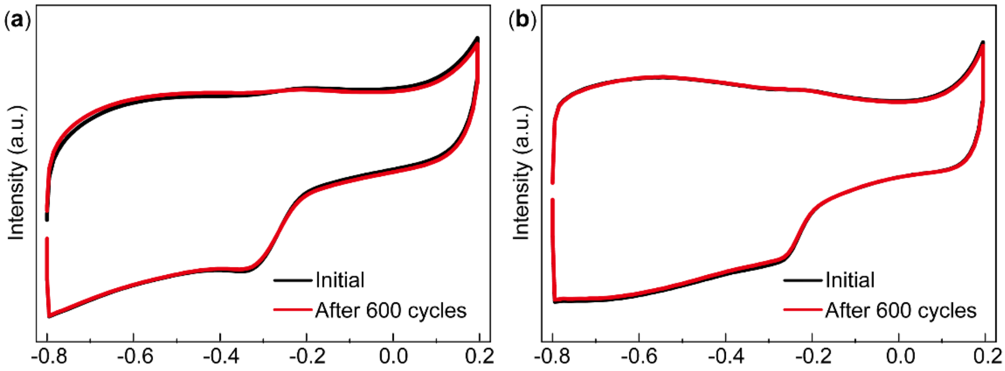

Figure S15. CV curves of (a) NiFe-LDH/rGO and (b) $Ni(OH)_2$/rGO at different cycle numbers in 1M KOH+1M CH_3OH at a scan rate of 50 mV s^{-1}.

REFERENCES

(1) Wu, S. L. *et. al*. Highly Dispersed Ultrafine Pt Nanoparticles on Reduced Graphene Oxide Nanosheets: In Situ Sacrificial Template Synthesis and Superior Electrocatalytic Performance for Methanol Oxidation. *ACS Appl. Mater. Interfaces* **7**, 22935-22940 (2015).

(2) Zhu, H. Y., Gu, C. D., Ge, X. & Tu, J. P. Targeted Growth of Pt on 2D Atomic Layers of Ni-Al Hydroxide: Assembly of the Pt/Exfoliated Ni-Al Hydroxide sheet/Graphene Composite as Electrocatalysts for Methanol Oxidation Reactions. *Electrochim. Acta* **222**, 938-945 (2016).

(3) Russo, P. A., Ahn, M., Sung, Y.-E. & Pinna, N. Improved electrocatalytic stability in ethanol oxidation by microwave-assisted selective deposition of SnO_2 and Pt onto carbon. *RSC Adv.* **3**, 7001-7008 (2013).

(4) Fan, Y., Liu, J. H., Lu, H. T., Huang, P. & Xu, D. L. Hierarchical structure SnO_2 supported Pt nanoparticles as enhanced electrocatalyst for methanol oxidation. *Electrochim. Acta* **76**, 475-479 (2012).

(5) Fu, X. Z., Liang, Y., Chen, S. P., Lin, J. D. & Liao, D. W. Pt-rich shell coated Ni nanoparticles as catalysts for methanol electro-oxidation in alkaline media, *Catal. Commun*. **10**, 1893-1897 (2009).

(6) Wang, C. Q., Jiang, F. X., Yue, R. R., Wang H. W. & Du, Y. Enhanced photo-electrocatalytic performance of Pt/RGO/TiO_2 on carbon fiber towards methanol oxidation in alkaline media. J. Solid State Electrochem **18**, 515-522 (2014).
